# Caffeic acid, but not ferulic acid, inhibits macrophage pyroptosis by directly blocking gasdermin D activation

**DOI:** 10.1002/mco2.255

**Published:** 2023-04-20

**Authors:** Mingjiang Liu, Dandan Liu, Chenglong Yu, Hua hao Fan, Xin Zhao, Huiwen Wang, Chi Zhang, Minxia Zhang, Ruonan Bo, Shasha He, Xuerui Wang, Hui Jiang, Yuhong Guo, Jingui Li, Xiaolong Xu, Qingquan Liu

**Affiliations:** ^1^ Beijing Key Laboratory of Basic Research With Traditional Chinese Medicine on Infectious Diseases Beijing Hospital of Traditional Chinese Medicine Capital Medical University Beijing China; ^2^ College of Veterinary Medicine Yangzhou University Yangzhou China; ^3^ Jiangsu Co‐innovation Center for Prevention and Control of Important Animal Infectious Diseases and Zoonoses Yangzhou China; ^4^ Beijing University of Chemical Technology Beijing China; ^5^ Beijing Chest Hospital Capital Medical University Beijing China

**Keywords:** caffeic acid, ferulic acid, gasdermin D, macrophage, pyroptosis, sepsis

## Abstract

Regulated pyroptosis is critical for pathogen elimination by inducing infected cell rupture and pro‐inflammatory cytokines secretion, while overwhelmed pyroptosis contributes to organ dysfunction and pathological inflammatory response. Caffeic acid (CA) and ferulic acid (FA) are both well‐known antioxidant and anti‐inflammatory phenolic acids, which resemble in chemical structure. Here we found that CA, but not FA, protects macrophages from both Nigericin‐induced canonical and cytosolic lipopolysaccharide (LPS)‐induced non‐canonical pyroptosis and alleviates LPS‐induced mice sepsis. It significantly improved the survival of pyroptotic cells and LPS‐challenged mice and blocked proinflammatory cytokine secretion. The anti‐pyroptotic effect of CA is independent of its regulations in cellular lipid peroxidation, mitochondrial function, or pyroptosis‐associated gene transcription. Instead, CA arrests pyroptosis by directly associating with gasdermin D (GSDMD) and blocking its processing, resulting in reduced *N‐*GSDMD pore construction and less cellular content release. In LPS‐induced septic mice, CA inhibits GSDMD activation in peritoneal macrophages and reduces the serum levels of interleukin‐1β and tumor necrosis factor‐α as the known pyroptosis inhibitors, disulfiram and dimethyl fumarate. Collectively, these findings suggest that CA inhibits pyroptosis by targeting GSDMD and is a potential candidate for curbing the pyroptosis‐associated disease.

## Graphical abstract

Liu et al. describe a function of caffeic acid (CA) in the regulation of lipopolysaccharide‐induced macrophage pyroptosis. CA prevents pro‐inflammatory cytokine secretion and pyroptotic cell death by directly blocking gasdermin D activation, and can obviously improve the survival rate of the sepsis mice to 80%. Ferulic acid, an analog of CA, fails to inhibit pyroptosis and improve mice survival rates.

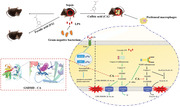

## 1 INTRODUCTION

Pyroptosis is a type of lytic cell death and a well‐regulated mechanism that defends the host against the insult of pathogenic microorganisms.^1^, ^2^, ^3^ Inflammasomes, the intracellular multiprotein complexes, are responsible for pyroptosis initiation and pathogens detection. Inflammasome activation requires two steps: priming and triggering. During the priming step, cytokines receptors or toll‐like receptors (TLRs) detect certain cytokines or microbial products and transduce these signals to nuclear factor‐κB (NF‐κB) or interferon regulatory factor 3 (IRF3) via the adapters MyD88 and TRIF, mediating the transcriptional preparation of inflammasome components and other pyroptosis‐associated proteins: NLR family pyrin domain containing 3 (NLRP3), pro‐caspase‐11, pro‐caspase‐1, and pro‐interleukin (IL)−1β.^4^, ^5^

The prepared NLRP3 recruits pro‐caspase‐1 in the presence of an apoptosis‐associated speck‐like protein containing a caspase recruitment domain (CARD) (ASC) to assemble NLRP3 inflammasome when encountering a spectrum of stimuli, including *Staphylococcus aureus*, *Listeria monocytogenes*, pore‐forming toxins, ATP, and crystals.^6^ At the inflammasome platform, pro‐caspase‐1 is processed into its active form.^6^ Activated caspase‐1 directly converts the precursors of proinflammatory cytokines, IL‐1β and IL‐18, into their mature forms. Caspase‐1 also cleaves gasdermin D (GSDMD) into its active *N‐*terminal fragment, which constructs pores in the plasma membrane to execute pyroptotic cell death and cellular contents release.^3^, ^7^, ^8^, ^9^, ^10^, ^11^ By contrast, cytosolic lipopolysaccharide (LPS) specifically activates non‐canonical inflammasome by promoting the activation of pro‐caspase‐11,^12^ which mediates pyroptosis by cleaving GSDMD without processing pro‐IL‐1β. Even so, in most macrophages, cytosolic LPS induces IL‐1β secretion, as the K^+^ efflux resulting from the *N‐*GSDMD pore can further activate NLRP3 inflammasome and caspase‐1.^5^, ^13^, ^14^

By recognizing external or internal threats, initiating inflammatory cascades, and killing infected host cells, pyroptosis plays a critical role in eliminating pathogens. Yet, excessive pyroptosis contributes to pathological responses, including abnormal coagulation, atherosclerosis, Alzheimer's disease, and so forth.^9^ Thus, it is necessary to research effective strategies for curbing uncontrolled cell pyroptosis. To date, some remarkable studies have been done in exploring endogenous or exogenous regulators of pyroptosis. For example, some pharmacological inhibitors, such as MCC950, VX765, and Glyburide, are identified to block pyroptosis by directly or indirectly inhibiting NLRP3 inflammasome.^15^, ^16^ Wedelolactone, cyclic adenosine monophosphate, and scutellarin are reported to block non‐canonical inflammasome by directly binding to caspase‐4/−11 or indirectly regulating the protein kinase A (PKA) pathway.^17^, ^18^, ^19^, ^20^ Disulfiram (DSF) and dimethyl fumarate (DMF) inhibit pyroptosis by limiting GSDMD processing or pore‐formation.^21^, ^22^, ^23^ Despite these progresses, the clinical use of these regulators is obstructed by their hepatotoxicity, unknown potential effects, or deficient research.^24^, ^25^

Recently, increasing efforts have been applied to the anti‐pyroptotic effect of natural compounds for their availability and relative safety.^26^, ^27^, ^28^ As component phenolic acids of lignin, caffeic acid (CA) and ferulic acid (FA) exist in all fruits and vegetables and are identified to be similar in lipoxygenase inhibition, oxidation resistance, and anti‐inflammation.^29^, ^30^, ^31^ While their role in pyroptosis is barely known.

In this work, we confirmed that CA could prevent macrophages from both nigericin‐induced canonical and cytosolic LPS‐induced non‐canonical pyroptosis. We further revealed that CA inhibited pyroptosis by directly blocking GSDMD activation, but not via its well‐known capacities in lipid peroxidation, mitochondrial protection, or pyroptosis‐associated gene transcription. In the LPS‐induced septic model, CA significantly promoted mice survival rates and suppressed activation of GSDMD in peritoneal macrophages (PMs). While FA exhibited no anti‐pyroptotic effect in vitro and in vivo.

## 2 RESULTS

### 2.1 CA, but not FA, dose‐dependently suppressed NLRP3 inflammasome‐mediated canonical pyroptosis

Among the canonical inflammasomes that activate caspase‐1, the NLRP3 inflammasome recognizes the widest range of stimuli, including pathogens, internal damage‐associated molecular patterns (DAMPs), and environmental irritants. Most of these stimuli activate NLRP3 inflammasome by inducing cell stress via multiple upstream signals: potassium efflux, lysosomal disruption, mitochondrial dysfunction, and so on.^4^

**FIGURE 1:**
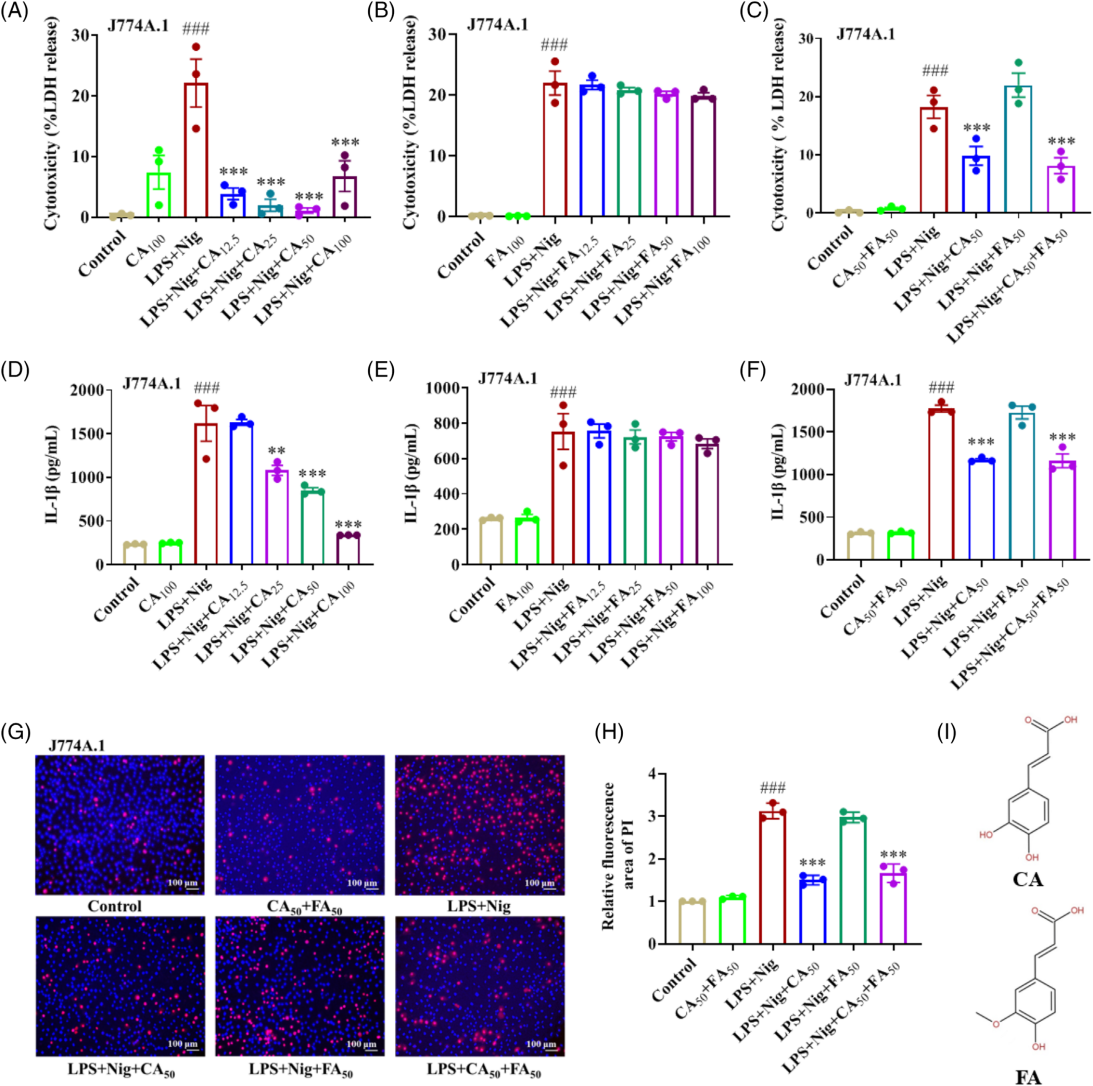
Caffeic acid (CA), but not ferulic acid (FA), dose‐dependently suppressed NLRP3 inflammasome‐mediated canonical pyroptosis. After being primed with 100 ng/ml lipopolysaccharide (LPS) for 4 h, J774A.1 cells were treated with 12.5, 25, 50, 100 µg/ml CA, FA, or their combination as indicated for 2 h and then with 10 µM Nigericin for 1 h. (A–C) The concentration of LDH in supernatants was analyzed by LDH cytotoxicity assay kits. (D–F) The secretion of IL‐1β in the culture medium was detected by ELISA kits. (G) The treated cells were stained by Hoechst 33342 (blue) and PI (red) and visualized under a fluorescence microscope. Scale bar: 100 µm. (H) The relative fluorescence area of PI. (I) The structures of CA and FA. The data are means ± standard error of the mean (SEM) (*n* = 3). **p < 0.01 and ***p < 0.001 versus the LPS + Nig group; *^###^p* < 0.001 versus the Control group. CA, caffeic acid; LPS, lipopolysaccharide; Nig, nigericin; FA, ferulic acid.

As a potassium‐proton ionophore antibiotic, Nigericin is a potent NLRP3 inflammasome inducer.^32^ J774A.1 macrophages resemble bone marrow‐derived macrophages (BMDMs) in terms of inflammasome activation.^33^ In this work, LPS‐primed J774A.1 cells were stimulated with 10 µM Nigericin to induce canonical pyroptosis. 12.5‐100 µg/ml CA treatment caused a concentration‐dependent reduction in releases of LDH and IL‐1β (Figure 1A,D), while 12.5‐100 µg/ml FA failed to block their secretion (Figure 1B,E). Meanwhile, FA showed no synergic effect with CA in LDH release, IL‐1β secretion, and PI staining (Figure 1C,F–H). These results indicate that CA markedly inhibits NLRP3 inflammasome‐dependent canonical pyroptosis. In contrast, FA fails to function as CA despite their similar chemical structures (Figure 1I).

### 2.2 CA, but not FA, blocked caspase‐11 inflammasome‐mediated non‐canonical pyroptosis

During cytosolic LPS‐induced pyroptosis, caspase‐11 plays a critical role in detecting the lipid A of cytosolic LPS via its CARD. The K^+^ efflux resulting from caspase‐11‐mediated GSDMD pore formation can further activate the NLRP3 inflammasome in most macrophages except RAW264.7, a cell line deficient in ASC expression.^34^, ^35^, ^36^ In this section, we employed RAW264.7 cells and BMDMs for our experiments to thoroughly explore how CA influences caspase‐11‐dependent non‐canonical pyroptosis.

**FIGURE 2:**
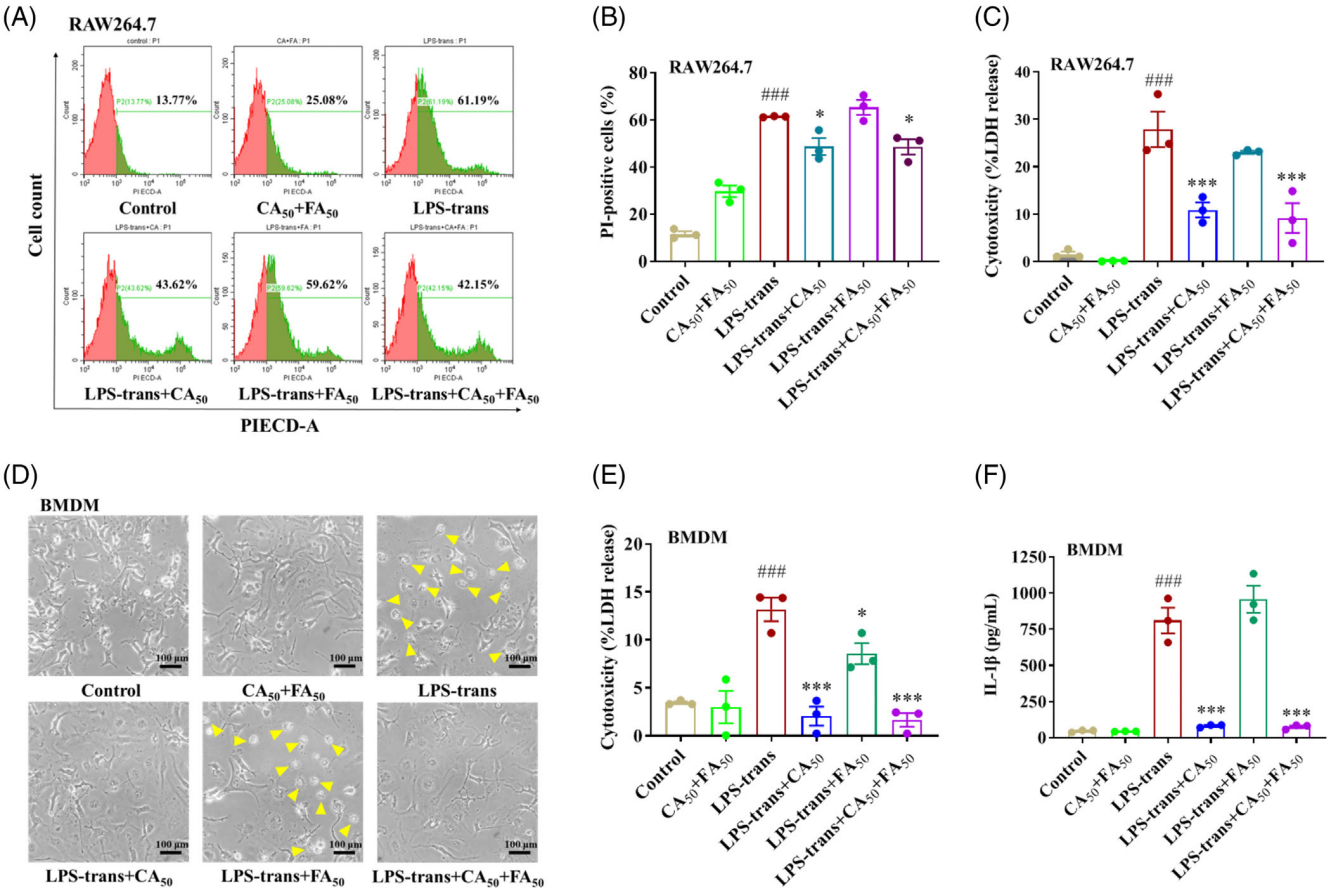
Caffeic acid (CA), but not ferulic acid (FA), blocked caspase‐11 inflammasome‐mediated non‐canonical pyroptosis. After being primed with 1 µg/ml lipopolysaccharide (LPS) for 6 h, RAW264.7 cells or bone marrow‐derived macrophages (BMDMs) were pretreated with 50 µg/ml CA, 50 µg/ml FA, or their combination for 2 h and then transfected with 2 µg/ml LPS using 0.25% FuGENE (v/v) for 16 h. (A) Cellular membrane permeability was evaluated by flow cytometry after PI staining. (B) The percentage of PI‐positive RAW264.7 cells was calculated. (C) The release of LDH by RAW264.7 cells was determined using the LDH cytotoxicity assay kit. (D) The treated BMDMs were visualized and photographed under an optical microscope. Yellow triangles indicate pyroptosis cells. The scale bar is 100 µm. (E) The LDH released by BMDMs was analyzed using the LDH cytotoxicity assay kit. (F) The concentration of IL‐1β in the culture medium of BMDMs was detected by ELISA kits. The data are means ± standard error of the mean (SEM) (*n* = 3). *p < 0.05 and ***p < 0.001 versus the LPS‐trans group. *^###^p* < 0.001 versus the Control group. LPS‐trans, LPS‐transfection.

For non‐canonical inflammasome activation, RAW264.7 cells and BMDMs were first primed with 100 ng/ml LPS and then transfected with 2 µg/ml LPS using 0.25% FuGENE HD. In agreement with its effect in canonical pyroptosis, CA markedly reduced LPS transfection‐induced LDH release, PI uptake, or cell rupture in RAW264.7 cells and BMDMs (Figure 2A–E), suggesting its protective role in membrane integrity. IL‐1β secretion in LPS‐transfected BMDMs was also potently suppressed by CA (Figure 2F). Consistent with the results in canonical pyroptosis, FA failed to exhibit anti‐pyroptotic effects in LPS‐transfected RAW264.7 cells and BMDMs.

Collectively, we concluded that CA performed well in restraining canonical and non‐canonical pyroptosis, while FA had no anti‐pyroptotic effect.

### 2.3 CA, but not FA, alleviated LPS‐induced sepsis in mice

**FIGURE 3:**
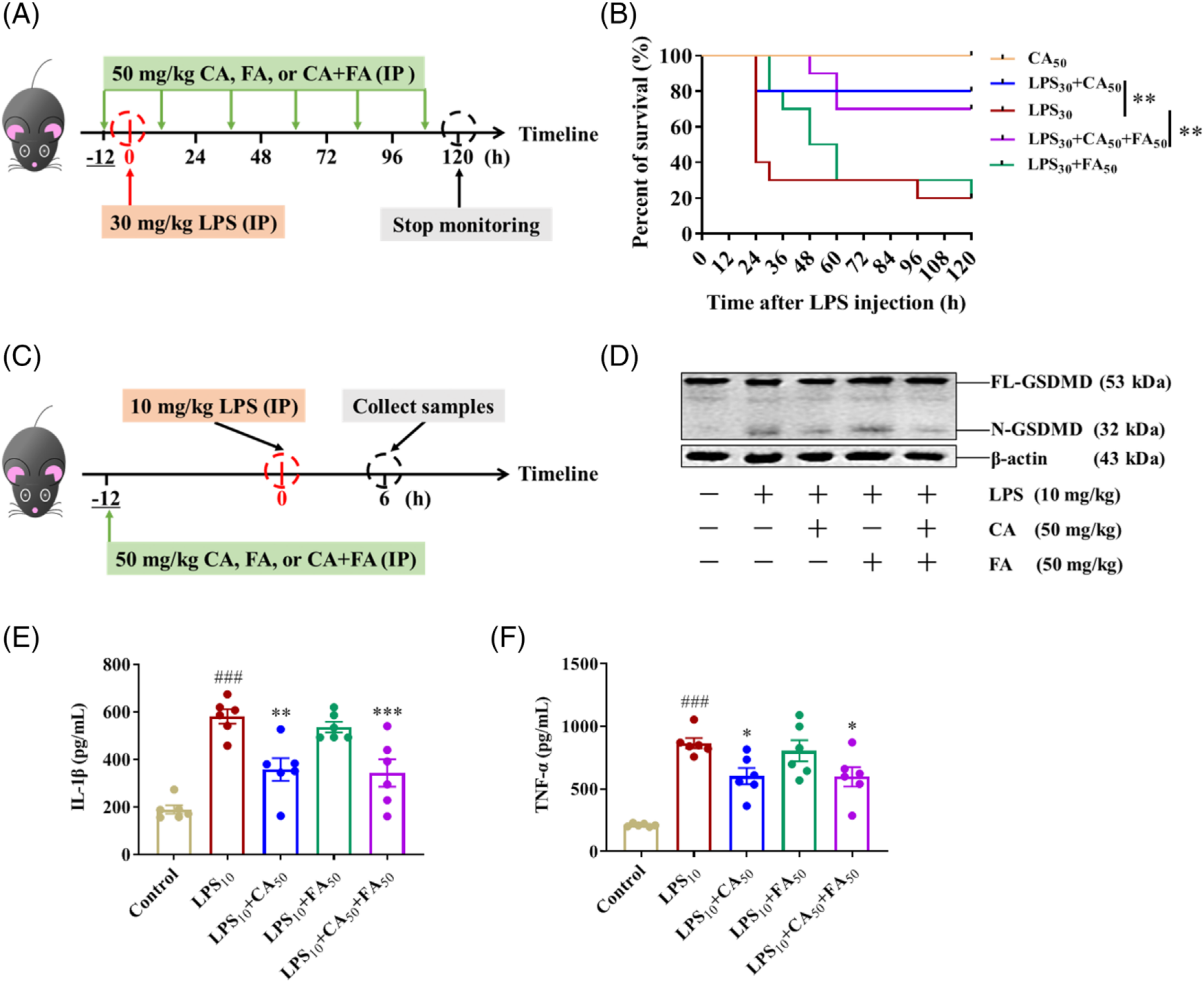
Caffeic acid (CA), but not ferulic acid (FA), alleviated lipopolysaccharide (LPS)‐induced sepsis in mice. (A and C) The schematic representations of treatment details. After pretreatments with 50 mg/kg CA, FA, or their combination by intraperitoneal injection, mice were challenged with 30 mg/kg or 10 mg/kg LPS for the indicated time. (B) After being challenged with 30 mg/kg LPS, the survival rate of mice was monitored every 6 h until 120 h. (D) After being challenged with 10 mg/kg LPS for 6 h, the peritoneal macrophages were collected and analyzed for gasdermin D (GSDMD) activation by immunoblotting. (E and F) After being challenged with 10 mg/kg LPS for 6 h, the concentrations of IL‐1β and TNF‐α in serum were analyzed by ELISA kits. Statistical analysis of the survival rate was performed using the log‐rank (Mantel‐Cox) test (*n* =  10). Statistical analysis of TNF‐α and IL‐1β concentration was performed using the one‐way analysis of variance (ANOVA) test (*n* = 6). The data are means ± standard error of the mean (SEM). *p < 0.05, **p < 0.01, and ***p < 0.001 versus the LPS group; *^###^p* < 0.001 versus the Control group. IP, intraperitoneal injection.

Circulating LPS initiates pyroptosis and causes lethal endotoxemia and septic cascade in the course of Gram‐negative bacteria infection, including *Escherichia coli*, *Salmonella typhimurium*, *Shigella flexnri*, and *Burkholderia thailandensi*s.^14^ In LPS‐induced mice sepsis, pretreatment of 50 mg/kg CA greatly promoted the survival rate of septic mice (Figure 3A,B) and reduced the serum concentrations of IL‐1β and tumor necrosis factor (TNF)‐α (Figure 3C,E,F). In the meantime, CA administration results in less GSDMD activation in PMs (Figure 3D). In comparison, FA failed to curb the death of septic mice, and it did not affect IL‐1β and TNF‐α serum levels.

### 2.4 The anti‐pyroptotic effect of CA is independent of its regulations in pyroptosis‐associated gene transcription, mitochondrion protection, and lipid peroxidation

**FIGURE 4:**
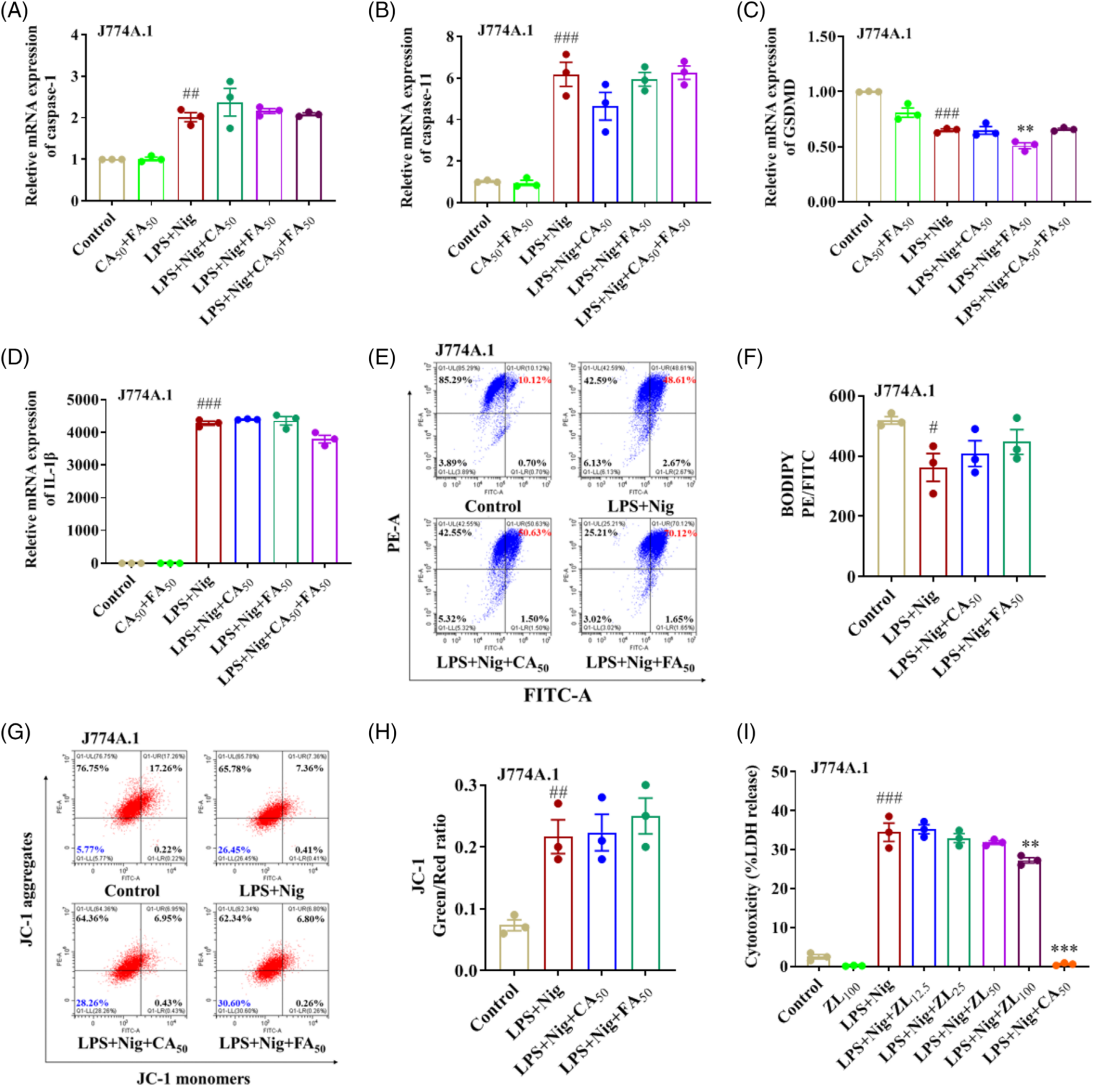
The anti‐pyroptotic effect of caffeic acid (CA) is independent of its regulatory activities on pyroptosis‐associated gene transcription, mitochondrion protection, and lipid peroxidation. (A‐H) J774A.1 cells primed with 100 ng/ml lipopolysaccharide (LPS) for 4 h were treated with 50 µg/ml CA, ferulic acid (FA), or their combination for 2 h, and then stimulated with 10 µM Nigericin for 1 h. (A–D) The mRNA expressions of *caspase‐1*, *caspase‐11*, *GSDMD*, and *IL‐1β* in treated J774A.1 cells were quantified by qRT‐PCR analysis. (E, F) Cellular lipid peroxidation was detected by BODIPY 581⁄591 C11 using flow cytometry and expressed as the ratio of red (reduced) to green (oxidized). (G and H) Mitochondrial membrane potential was detected by JC‐1 using flow cytometry and indicated by the ratio of green (JC‐1 monomers) to red (JC‐1 aggregates). (I) J774A.1 cells primed with 100 ng/ml LPS for 4 h were treated with the indicated concentration of Zileuton or CA for 2 h and then stimulated with 10 µM Nigericin for 1 h. The LDH release in the supernatant was measured by the LDH cytotoxicity assay kit. The data are means ± standard error of the mean (SEM) (*n* = 3). *p < 0.05, **p < 0.01, and ***p < 0.001 versus the LPS + Nig group; *^#^p* < 0.05, *^##^p* < 0.01 and *^###^p* < 0.001 versus the Control group. ZL, Zileuton.

Prior to inflammasome activation, LPS priming is a critical step for the NF‐κB‐ and IRF3‐mediated transcriptional preparation of inflammasome components.^37^, ^38^ Previous works have reported that CA and FA could inhibit LPS‐induced NF‐κB activation and inflammatory responses.^5^, ^12^, ^39^, ^40^, ^41^ To explore the effects of CA or FA on gene transcription during pyroptosis, we primed cells with LPS and detected the mRNA expressions of *caspase‐1*, *IL‐1β*, *caspase‐11* and *GSDMD* in Nigericin‐induced pyroptotic cells, and we observed that they have little impact on them (Figure 4A‐4D).

Lipid peroxidation and mitochondrion dysfunction are critical features and upstream signals of pyroptosis.^9^, ^42^ Lipoxygenase has been shown to induce lipid peroxidation and involves pyroptosis.^43^, ^44^ CA and FA are well‐known natural antioxidants, mitochondrion protectors, and lipoxygenase inhibitors.^42^, ^45^, ^46^ To clarify the role of CA and FA in antioxidation and mitochondrion protection during pyroptosis, we detected the lipid peroxidation and mitochondrial membrane potential of the pyroptotic cells, and we found that both CA and FA have no impact on them (Figure 4E–H). Further, to identify if lipoxygenase inhibition functions in blocking pyroptosis, we tested the anti‐pyroptotic effect of another lipoxygenase inhibitor Zileuton. The results revealed that 12.5–50 µM Zileuton did not influence the LPS/Nigericin‐induced LDH release in J774A.1 cell. 100 µM Zileuton suppressed LDH release, while its effect is far less than 50 µg/ml CA (Figure 4I). Thus, we concluded that CA and FA have little influence on proptosis‐associated gene transcription, lipid peroxidation, and mitochondrion dysfunction during pyroptosis.

### 2.5 CA inhibited macrophage pyroptosis by blocking the formation of *N‐*GSDMD pores

**FIGURE 5:**
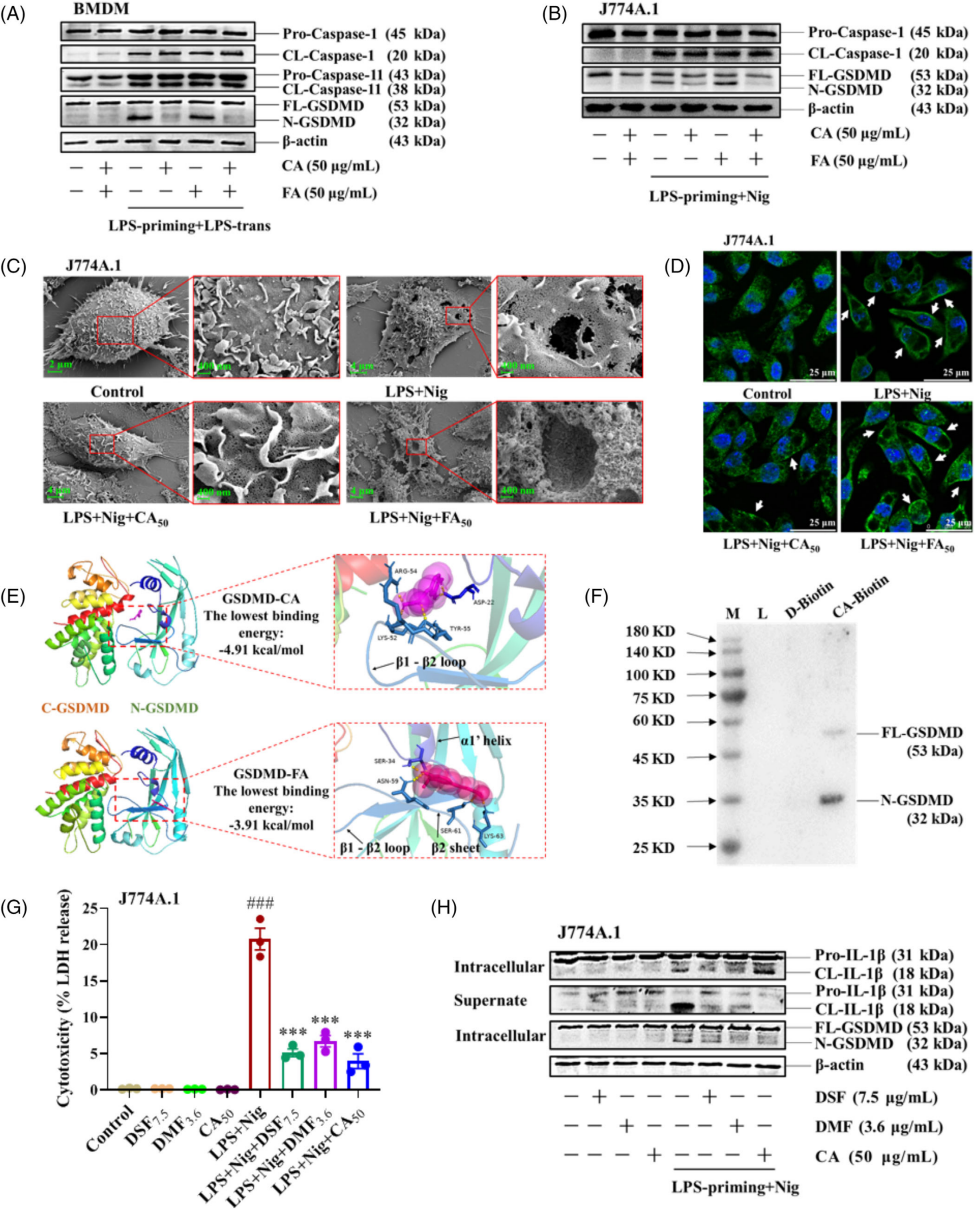
Caffeic acid (CA) inhibited macrophage pyroptosis by blocking the formation of *N‐*gasdermin D (GSDMD) pores. (A, B) Lipopolysaccharide (LPS)‐primed bone marrow‐derived macrophages (BMDMs) were treated with 50 µg/ml CA or ferulic acid (FA) for 2 h by replacing the culture medium and then were transfected with 2 µg/ml LPS using 0.25% FuGENE (v/v) for 16 h. LPS‐primed J774A.1 cells were treated with 50 µg/ml CA or FA for 2 h before 10 µM Nigericin stimulation for 1 h. The cell pellets were collected and the activation of critical proteins was analyzed by western blot assay. (C) The cell micromorphology was observed and pictured under the standard error of the mean (SEM). (D) The aggregation of *N‐*GSDMD (green) was photographed under a laser confocal microscope using the immunofluorescence assay. White arrows indicate the aggregation of *N‐*GSDMD in the cell membrane. (E) The binding poses of CA or FA to GSDMD were calculated by Autodock. The ligand‐receptor interaction interfaces were highlighted by red dotted boxes. The close‐up views of them were shown aside, in which the hydrogen bonds were indicated as yellow dotted lines, and the interacted residues were colored in blue. (F) The GSDMD‐CA binding was confirmed by a biotin affinity pull‐down assay. The pulled‐down proteins were detected by immunoblotting. (G and H) LPS‐primed J774A.1 cells were treated with 7.5 µg/ml DSF, 3.6 µg/ml DMF, or 50 µg/ml CA for 2 h before 10 µM Nigericin stimulation. The cytotoxicity was determined by the LDH cytotoxicity assay kit. The cleaved IL‐1β in the cytoplasm and supernatant were analyzed by western blot assay. ***p < 0.001 versus the LPS + Nig group; *^###^p* < 0.001 versus the Control group. DSF, disulfiram; DMF, dimethyl fumarate.

The above results suggest that it's CA, but not FA, that potently inhibits both canonical and non‐canonical pyroptosis. To determine the action target of CA, we assessed its impact on the activation of proinflammatory caspases and GSDMD. Immunoblot results indicated that CA did not influence the cleavage of caspase‐1 and caspase‐11 in LPS/Nigericin‐treated J774A.1 cells and LPS‐transfected BMDMs, while the *N‐*GSDMD formations were significantly reduced by CA in both cells (Figure 5A,B). Under an electron microscope, the LPS/Nigericin‐induced cell rupture and pore formation were significantly suppressed by CA (Figure 5C). Using immunofluorescence assay, we found that CA markedly inhibited the aggression of *N‐*GSDMD in the plasma membrane (Figure 5D). Thus, we speculated that CA inhibited macrophage pyroptosis by blocking *N‐*GSDMD pore formation. In comparison, FA had no such effects (Figure 5A–D).

To figure out the interaction of CA with GSDMD, we used Autodock, a suite of automated docking tools, to calculate the best binding conformation and the corresponding lowest binding energy. CA interacted with the *N‐*GSDMD by forming hydrogen bonds with the ASP22, LYS52, TYR55, and ARG54 residues located at or near the α1 helix and β1‐β2 loops, and their lowest binding energy was −4.91 kcal/mol. In comparison, FA bound to the α1’ helix and β2 strand of *N‐*GSDMD by forming hydrogen bonds with the SER34, ASN59, SER61, and LYS63 residues with a −3.91 kcal/mol binding energy (Figure 5E). We also assessed the binding of CA and GSDMD by biotin affinity pull‐down assay, and we found that both full‐length GSDMD and *N‐*GSDMD were pulled down by CA‐biotin (Figure 5F).

Next, we compared the cytotoxicity in the presence of CA, DSF, and DMF. As predicted, CA ameliorated cytotoxicity as well as other GSDMD inhibitors (Figure 5G). Then the activation and secretion of IL‐1β were assessed by immunoblotting. CA treatment had no apparent effect on the maturation of IL‐1β, but it substantially blocked the secretion of IL‐1β in the culture supernatant (Figure 5H). These results further confirm that CA inhibited macrophage pyroptosis by directly blocking GSDMD activation, but not the processing of caspases and IL‐1β.

### 2.6 CA, like DSF and DMF, can protect mice from LPS‐induced sepsis by inhibiting macrophage pyroptosis

**FIGURE 6:**
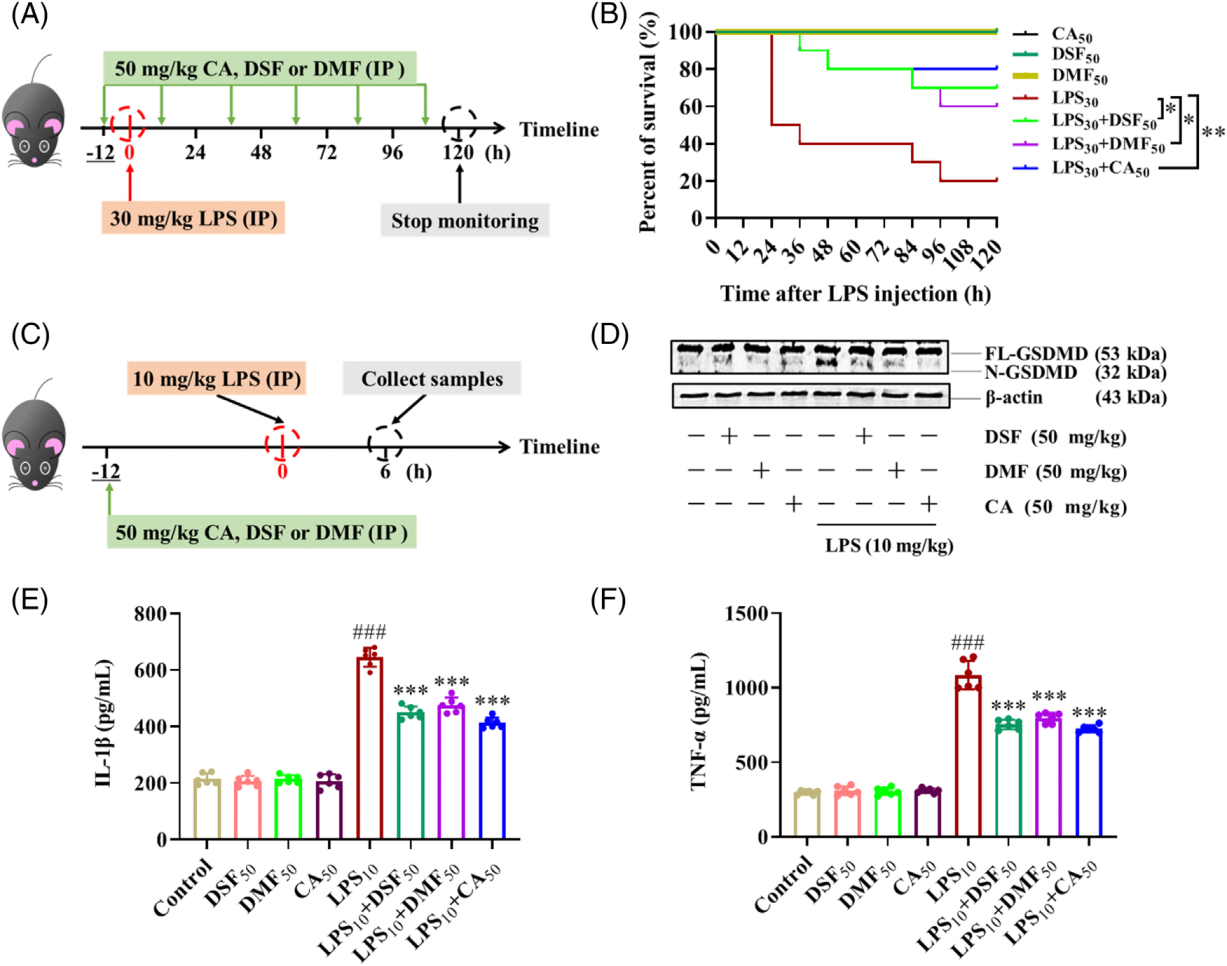
Caffeic acid (CA), like DSF and DMF, can protect mice from lipopolysaccharide (LPS)‐induced sepsis by inhibiting macrophage pyroptosis. (A, C) The schematic representation of treatment details. After 12 h pretreatments with 50 mg/kg CA, DSF, or DMF by intraperitoneal injection, mice were challenged with 30 mg/kg or 10 mg/kg LPS for the indicated time. (B) After being challenged with 30 mg/kg LPS, the survival rate of mice was monitored every 6 h until 120 h. (D) After being challenged with 10 mg/kg LPS for 6 h, the peritoneal macrophages were collected and analyzed for gasdermin D (GSDMD) activation by immunoblotting. (E, F) After being challenged with 10 mg/kg LPS for 6 h, the concentrations of IL‐1β and TNF‐α in serum were analyzed by ELISA kits. Statistical analysis of the survival rate was performed using the log‐rank (Mantel‐Cox) test (*n* =  10). Statistical analysis of TNF‐α and IL‐1β concentration was performed using the one‐way analysis of variance (ANOVA) test (*n* = 6). The data are means ± SEM. *p < 0.05, **p < 0.01, and ***p < 0.001 versus the LPS group; *^###^p* < 0.001 versus the Control group. IP, intraperitoneal injection.

In vivo, we compared the protective roles of CA with DSF and DMF in septic mice. As expected, all of these inhibitors improved the survival rate of septic mice (Figure 6A,B) and reduced the serum IL‐1β and TNF‐α (Figure 6C,E,F). PMs gathered 6 h post‐LPS challenge showed a low level of cleaved GSDMD with CA treatment (Figure 6D). Collectively, these data indicated that the inhibition effect of CA on macrophage pyroptosis plays an important role in alleviating mice sepsis, and its efficacy is similar to DSF and DMF.

## 3 DISCUSSION

Pyroptosis is a well‐regulated cell death that is initiated by inflammasomes, mediated by inflammatory caspases, and executed by pore‐forming GSDMD.^47^ Although it is critical for combatting pathogens, aberrant activation of pyroptosis has been implicated in detrimental pathological damages.^7^ Thus, it gained a lot of attention to explore exogenous and endogenous down‐regulatory factors of pyroptosis.

There are classical and non‐canonical pathways of pyroptosis. To clarify which steps in pyroptosis pathways were inhibited by CA, we employed three types of mice macrophages (J774A.1, BMDM, and RAW264.7) for our in vitro experiments. J774A.1, a cell line that resembles BMDM in terms of inflammasome activation, was primed with LPS and stimulated with Nigericin, a potassium‐proton ionophore, for NLRP3 inflammasome activation.^32^, ^33^ BMDMs and RAW264.7 cells were transfected with LPS using FuGENE reagent for caspase‐11 inflammasome activation. Unlike LPS‐transfected BMDM, caspase‐11 activation cannot trigger NLRP3 inflammasome via *N‐*GSDMD pore formation in RAW264.7 for lack of ASC expression.^32^, ^33^ In these cell lines, we observed that CA efficiently inhibited both NLRP3 inflammasome‐ and caspase‐11 inflammasome‐mediated pyroptosis: LPS/Nigericin‐ and LPS transfection‐induced LDH release and IL‐1β secretion were all dose‐dependently suppressed by CA, and CA significantly prevented J774A.1 and BMDM from LPS/Nigericin‐ and LPS transfection‐induced PI‐intake and characteristic pyroptotic morphological changes. FA did not function in these processes and showed no synergistic effect with CA despite their similar structures. Collectively, these results enlightened us that CA exhibited a remarkable regulatory effect in pyroptosis.

Triggering of pyroptosis requires two steps: priming and activation. During the priming step, pyroptosis‐associated genes, including *NLRP3*, *caspase‐1*, *IL‐1β*, and *caspase‐11*, are transcribed in a TLRs‐dependent manner.^48^ These genes were translated into related proteins which were revealed to be activated by cellular lipid peroxidation and mitochondrion dysfunction and trigger pyroptosis.^9^, ^42^ Notably, CA and FA are identified to be inhibitors in the TLR4‐NF‐κB pathway,^41^, ^49^ lipid peroxidation,^43^ and mitochondrial membrane potential damage,^50^ in previous reports. In this work, we primed cells with LPS to initiate TLR4‐dependent gene transcription. While, to our surprise, both CA and FA have no impact on the pyroptotic gene expression, lipid peroxidation, or the damage of mitochondrial membrane potential in pyroptotic cells. That might explain why FA failed to curb pyroptosis, although it resembles CA in structure and aforementioned activities.^31^, ^51^, ^52^

Lipoxygenases (LOXs) are iron‐containing enzymes that catalyze the arachidonic acid into hydroperoxy eicosatetraenoic acid (HPETE) and leukotrienes, and they are closely related to inflammation, lipid peroxidation, as well as programmed cell death.^43^, ^44^, ^53^ As a potent LOX inhibitor, CA was previously reported to prevent H_2_O_2_‐ and nonsteroidal anti‐inflammatory drug‐induced programmed cell death.^54^, ^55^ FA is also described as a LOX inhibitor in previous reports.^56^, ^57^ To explore the role of LOX inhibition in pyroptosis and the connection between the anti‐pyroptotic and anti‐LOX activity of CA, we employed another LOX inhibitor, Zileuton.^58^ We found that 12.5–50 µM Zileuton failed to suppress the LPS/Nigericin‐induced release of LDH. Although 100 µM Zileuton inhibited LDH release, its effect was far weaker than 50 µg/ml CA. These results implied that CA did not inhibit pyroptosis via its LOX inhibitory activity.

As mentioned above, CA markedly blocked both canonical and non‐canonical pyroptosis, and it is independent of its well‐known activities. To unveil the action mechanism of CA, we investigated its impact on the activation of caspase‐1, caspase‐11, and GSDMD. Our immunoblot assay suggested that CA greatly blocked *N‐*GSDMD formation in both LPS/Nigericin‐induced J774A.1 cells and LPS‐transfected BMDMs, without affecting the activation of caspase‐1 and caspase‐11. Immunofluorescence results indicated that CA reduced the aggregation of *N‐*GSDMD in the plasma membrane of pyroptotic cells. Consistently, under an optical microscope and scanning electron microscopes, we found that CA actually ameliorated the characteristic cell rupture and membrane pore formation of pyroptotic cells. These results enlightened us that CA directly inhibited pyroptotic cell death by restraining the formation of *N‐*GSDMD membrane pores.

The discovery that CA might downregulate pyroptosis by blocking GSDMD activation encouraged us to explore its possible interaction mode with GSDMD. GSDMD possesses an auto‐inhibitory conformation, in which the C‐terminal inhibitory domain binds to the *N‐*terminal pore‐forming domain and blocks its lipid‐binding surface.^59^, ^60^ There are two interaction sites that associate the *N‐*GSDMD with the C‐GSDMD. First, the α5, α8, and α12 helices of C‐GSDMD bind to the α1 helix and β1‐β2 loop of *N‐*GSDMD. This linker region harbors the specific caspase cleavage site, the _273_LLSD_276_ sequence, and the lipid‐binding sites, the α1 helix and β1‐β2 loop.^61^, ^62^, ^63^, ^64^ Secondly, the α9 and α11 helices of C‐GSDMD interact with the α4 helix of *N‐*GSDMD.^59^, ^65^ Via Autodock tools, we observed that CA interacted with GSDMD by forming hydrogen bonds with ASP22, LYS52, TYR55, and ARG54 residues located at the β1‐β2 loop of *N‐*GSDMD with a −4.91 kcal/mol binding energy, which might explain its down‐regulatory role in GSDMD activation. By comparison, with a −3.91 kcal/mol binding energy, FA binds to the SER34, ASN59, SER61, and LYS63 residues located at the α1’ helix and β2 sheet that are distant from the intramolecular domain interface. Biotin affinity pull‐down assay further confirmed the GSDMD‐CA interaction. As expected, CA inhibited pyroptotic cell death as well as other known pyroptosis inhibitors, DSF and DMF that were reported to target GSDMD.^21^, ^22^, ^23^ According to the immunoblotting, the activation of IL‐1β in the cytoplasm was not influenced by CA, but mature IL‐1β was barely detected in supernatant, which suggested that CA directly targeted GSDMD. Although the interaction and binding mode of CA to GSDMD were explained, the exact binding principle needs further explanation.

During gram‐negative bacteria infection, caspase‐11 activated by cytosolic LPS cleaves GSDMD, and the released *N‐*GSDMD mediates both pyroptosis and NLRP3 inflammasome activation, which promotes sepsis.^14^ Thus, GSDMD has gained a lot of attraction as a potential therapeutic target for the treatment of pyroptosis‐associated diseases, including LPS‐induced endotoxemia and sepsis.^66^, ^67^, ^68^ In the present work, we testified the in vivo effect of CA in LPS‐induced septic mice as a pyroptosis inhibitor. And we found that it significantly reduced IL‐1β and TNF‐α in serum and promoted mice survival as other reported GSDMD inhibitors. Further, the activation of GSDMD in PMs was reduced by CA administration in LPS‐induced septic mice. While FA had no such activities. Collectively, we concluded that CA could alleviate LPS‐induced sepsis by inhibiting pyroptosis.

## 4 CONCLUSION

**FIGURE 7:**
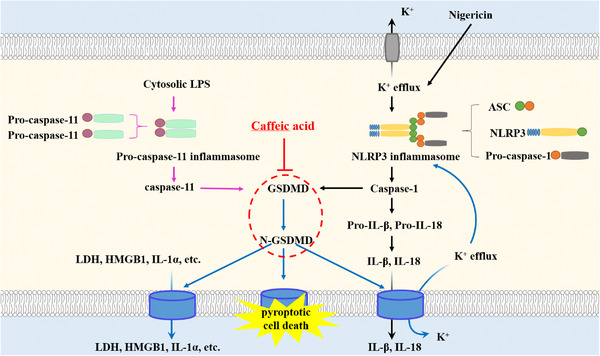
The schematic overview of the proposed anti‐pyroptotic mechanism of caffeic acid (CA). Canonical NLRP3 inflammasome can be activated by a wide range of stimuli that induce potassium ions efflux, lysosomal disruption, and mitochondrial dysfunction. As a potassium‐proton ionophore antibiotic, Nigericin is a potent NLRP3 inflammasome inducer, which leads to the assembly of NLRP3 inflammasome and activation of caspase‐1. Active caspase‐1 directly converts the pro‐inflammatory cytokines, pro‐IL‐1β and pro‐IL‐18, into their mature forms. Additionally, caspase‐1 cleaves gasdermin D (GSDMD) and liberates the pore‐forming *N‐*GSDMD to execute pyroptotic cell death and release of cellular contents. In comparison to NLRP3 inflammasome, non‐canonical inflammasome is specifically activated by cytosolic lipopolysaccharide (LPS), which promotes the activation of caspase‐11. Caspase‐11 also cleaves GSDMD and mediates lytic cell death, while it does not directly process pro‐inflammatory cytokines. Even so, in most macrophages, caspase‐11‐mediated GSDMD pore formation can lead to NLPR3‐dependent maturation and secretion of IL‐1β and IL‐18 by inducing potassium ions efflux. CA inhibits both canonical and non‐canonical pyroptotic cell death and IL‐1β secretion by blocking the activation of GSDMD, the key pyroptosis executor protein.

Collectively, CA prevents pro‐inflammatory cytokine secretion and pyroptotic cell death by directly blocking GSDMD activation (Figure 7) and alleviates LPS‐induced sepsis in mice. FA, an analog of CA, fails to inhibit pyroptosis. Although the effect of CA in specific disease models needs further illustrations, we demonstrate that it is a potential candidate for inhibiting pyroptosis‐associated disease.

## 5 MATERIALS AND METHODS

### 5.1 Reagents and antibodies

CA (110885) and FA (110773) were bought from the National Institutes for Food and Drug Control (Beijing, China). DSF (HY‐80240/CS‐2209), DMF (HY‐17363/CS‐0909), and Biotin (HY‐B0511) were bought from MedChemExpress (New Jersey, USA). Streptavidin agarose beads were bought from Yeasen Biotech (Shanghai, China). LPS from *E. coli* O55:B5 (L2880) was bought from Sigma Aldrich (St. Louis, MO, USA). Fetal bovine serum (FBS, 10091148) and opti‐Dulbecco's modified Eagle medium (DMEM, 31985‐070) was bought from Gibico (Waltham, MA, USA). Ammonium‐Chloride‐Potassium (ACK) lysis buffer (R1010) and Hoechst 33342/PI double stain kit (CA1120‐100T) were bought from Solarbio (Beijing, China). Recombinant murine macrophage colony‐stimulating factor (M‐CSF, 315‐02‐10 µg) was bought from PeproTech (United States). FuGENE HD (E2311) was bought from Promega (Madison, WI, USA). Nigericin (tlrl‐nig) was bought from Invivogen (San Diego, CA, USA). Bicinchoninic acid assay (BCA) protein assay kit (P0012S), LDH cytotoxicity assay kit (C0017), and mitochondrial membrane potential assay kit with JC‐1 (C2006) were bought from Beyotime (Shanghai, China). ELISA kits for murine IL‐1β (RK00006) and TNF‐α (RK00027) were bought from ABclonal (Wuhan, China). BODIPY 581⁄591 C11 (D3861) was bought from Invitrogen (Carlsbad, USA). RNA isolator total RNA extraction reagent (R401‐01), HisciptIIQRT SuperMix (R123‐01), and ChamQTM SYBR qPCR Master Mix (Q311‐02) were bought from Vazyme Biotech (Nanjing, China). Polyvinylidene difluoride (PVDF) membrane was bought from Merck Millipore (MA, USA). Antibodies against GSDMD (209845) and caspase‐11 (180673) were bought from Abcam (Cambridge, UK). Antibodies against β‐actin (4970) and caspase‐1 (24232) were bought from Cell Signaling Technology (Danvers, MA, USA). Antibodies against IL‐1β (A11370) were bought from ABclonal (Wuhan, China). Alexa Fluor 488 AffiniPure goat anti‐mouse IgG (FMS‐Msaf48801) was bought from Fcmacs Biotech (Nanjing, China). Biotin was bought from MedChemExpress (New Jersey, USA).

### 5.2 Mice

Female C57BL/6 mice aged 6–12 weeks were purchased from the Comparative Medicine Centre of Yangzhou University (SCXK‐2017‐0007, Jiangsu, China) and kept under controlled conditions (20 ± 2°C, 60% humidity, 12 h light/dark) in experimental animal room with free access to standard laboratory chow and sterile water (SYXK‐2017‐0044). All animal experimental protocols were ethically approved by the Animal Care and Use Committee of Yangzhou University (202103‐008).

### 5.3 LPS‐induced mouse sepsis model

For the survival study, C57BL/6 mice were intraperitoneally pretreated with 50 mg/kg CA, 50 mg/kg FA, 50 mg/kg CA + 50 mg/kg FA, 50 mg/kg DSF, 50 mg/kg DMF, or an equal volume of phosphate‐buffered saline (PBS) for 6 h before 30 mg/kg LPS intraperitoneal injection. The survival rate was monitored and recorded every 12 h post‐LPS administration. For mechanism analysis, 50 mg/kg CA, 50 mg/kg DSF, 50 mg/kg DMF, or an identical volume of PBS were administered intraperitoneally for 6 h before the 10 mg/kg LPS intraperitoneal challenge. 12 h after the LPS challenge, mice were anesthetized with pentobarbital sodium and sacrificed by cervical dislocation for collections of serum and PMs.

### 5.4 Cell culture

Bone marrow‐derived macrophages (BMDMs) were generated as previously described.^69^ Briefly, 6−12 week old C57BL/6 mice were anesthetized, executed by cervical dislocation, and dissected for tibias and femur stripping. Under an aseptic environment, bone marrow in the tibias and femurs was flushed out using 5 ml syringes until the medullary cavity turned white. After lysis by ACK buffer, the flushed‐out bone marrow was washed with PBS, dispersed by DMEM medium containing 20 ng/ml M‐CSF, and subsequently plated in 6‐wells plates with 1×10^6^ cells per well. The DMEM containing 20 ng/ml M‐CSF was half‐replaced every 3 days for 6 days. The PMs of mice in animal experiments were harvested by lavage of the peritoneal cavity after serum collection and cervical dislocation according to previous reports.^23^ The PM suspension was centrifuged, and the PM pellets were lysed for immunoblot assay.

Murine monocyte‐macrophage cell lines RAW264.7 and J774A.1 were purchased from China's national infrastructure of cell line resource and cultured in DMEM supplemented with 10% (v/v) FBS at 37°C and 5% CO_2_.

### 5.5 Inflammasome activation assay

To activate NLRP3 inflammasome, J774A.1 cells were primed with 100 ng/ml LPS for 4 h and stimulated with 10 µM Nigericin for 1 h. For cytosolic LPS‐induced non‐canonical inflammasome activation, BMDM and RAW264.7 cells were primed with 1 µg/ml LPS for 6 h and transfected with 2 µg/ml LPS using 0.25% FuGENE HD (v/v) for 16 h by replacing the culture medium. For drug administration assay, 50 µg/ml CA or FA was administered for 2 h after LPS‐priming, following which the inflammasome activators were added.

### 5.6 LDH cytotoxicity assay

LDH in the supernatant was quantified according to the manufacturer's instructions. Briefly, the culture mediums were collected and incubated with an equal volume of LDH assay working cocktails in the dark at room temperature for 25 min. The absorbance was measured by a microplate reader (BioTek, Winooski, VT, USA) at a wavelength of 490 nm.

### 5.7 PI incorporation assay

Collected cells were resuspended in 1 ml staining buffer containing 5 µL Hoechst and 5 µL PI. After 20 min of incubation at room temperature, cells were washed and centrifuged for determinations by flow cytometry (CytoFLEX, Beckman, China) or fluorescence microscope (AXIOVERT A1, Carl Zeiss, Germany).

### 5.8 Enzyme‐linked immunosorbent assay (ELISA)

For IL‐1β or TNF‐α concentration measurements, culture mediums or serums were collected and examined according to the manufacturer's instructions. The absorbance was measured by a microplate reader (BioTek, Winooski, VT, USA) at a wavelength of 450 nm.

### 5.9 Quantitative real‐time polymerase chain reaction

According to the manufacturer's instructions, the total RNA of cells was generated by TRIzol reagent and quantified by a spectrophotometer (Thermo Fisher Scientific Inc, MA, USA). Note that, 1 µg of total RNA was used to synthesize cDNA for subsequent quantitative real‐time polymerase chain reaction (qRT‐PCR) with SYBR Green PCR kits in the CFX96 RT‐PCR Detection System (Bio‐Rad Laboratories Inc, MA, USA). β‐actin was used as a normalization control, and the relative expression level was calculated by 2^−ΔΔCt^. The cycle conditions were as follows: an initial denaturation step at 95°C for 5 min, 40 cycles of denaturation for 10 s at 95°C, and annealing and extension for 30 s at 60°C. Primers for qRT‐PCR were bought from Tsingke Biotechnology (Beijing, China). Their sequences were listed below in Table 1.

**TABLE 1:** Gene‐specific oligonucleotide primer used for quantitative real‐time polymerase chain reaction (qRT‐PCR).

**Item**	**Accession number** **^a^**	**Primer sequence (5′–3′)**	**Product size (bp)**
*β‐actin*	NM_007393.5	F: TGTTACCAACTGGGACGACA R: TCTCAGCTGTGGTGGTGAAG	392
*IL‐1β*	NM_008361.4	F: CCTGCAGCTGGAGAGTGTGGAT R: TGCTCTGCTTGTGAGGTGCTG	150
*Caspase‐1*	NM_009807.2	F: GGCACATTTCCAGGACTGACTG R: GCAAGACGTGTACGAGTGGTTG	125
*Caspase‐11*	Y13089.1	F: ACGATGTGGTGGTGAAAGAGGAGC R: TGTCTCGGTAGGACAAGTGATGTGG	435
*GSDMD*	NM_026960.4	F: GCGATCTCATTCCGGTGGACAG R: TTCCCATCGACGACATCAGAGAC	208

^a^ Primers were designed from the published sequences in the GenBank database under the indicted accession numbers.

^b^ F = forward primer; R = reverse primer.

### 5.10 Lipid peroxidation measurement

Cells were collected by centrifugation and incubated with 10 µM C11 BODIPY 581/591 for 30 min. The fluorescence was recorded by flow cytometry (CytoFLEX, Beckman, China) in 581/591 nm (red) for reduced dye and 488/510 nm (green) for oxidized dye. The ratio of red to green was output as the indicator of lipid peroxidation.

### 5.11 Mitochondrial membrane potential measurement

After trypsinization and centrifugation, cells were incubated with 2 µM JC‐1 in a 37°C incubator for 20 min. Stained cells were washed twice with precooled assay buffer and analyzed by flow cytometry (CytoFLEX, Beckman, China). The ratio of green to red was output as the indicator of mitochondrial membrane potential damage.

### 5.12 Western blotting

The total cell proteins and supernatant proteins were prepared and quantified using a BCA protein assay kit. The obtained proteins were mixed with a 4X loading buffer and boiled for 5 min. 20 µg of the denatured proteins were resolved by sodium dodecyl sulfate‐polyacrylamide gel electrophoresis (SDS‐PAGE) and transferred to PVDF membranes. After being blocked by skimmed milk, the membranes were incubated with specific primary antibodies overnight and secondary antibodies for 2 h. Stripes were detected by Chemidoc XRS (Bio‐Rad, Marnes‐la‐Coquette, France) using ECL. Densitometric values of immunoblots were quantified using ImageJ and normalized against β‐actin to correct for differences in protein loading.

### 5.13 Immunofluorescence assay

To monitor the GSDMD aggregation in the plasma membrane, LPS/Nigericin‐induced J774A.1 cells were fixed with 4% paraformaldehyde for 15 min, permeabilized by 0.1% Triton X‐100 for 20 min, and blocked by 5% BSA for 1 h. After washing with PBS, cells on coverslips were immersed in GSDMD antibody overnight and fluorescent‐conjugated secondary antibodies for 1 h. The nuclei were stained with 2‐(4‐amidinophenyl)‐6‐indolecarbamidine dihydrochloride for 5 min. Cells were visualized and imaged under confocal microscopy (TCS SP8 STED, Leica, Germany).

### 5.14 Scanning electron microscope

J774A.1 cells were seeded on climbing slides and treated with LPS/Nigericin to induce NLRP3 inflammasome activation, and then cells were fixed in 2.5% glutaraldehyde overnight and washed with PBS. After dehydration in gradient ethanol, the slides were dried by CPD‐300 critical point dryer and attached to a sample holder for observation under a field emission scanning electron microscope system (GeminiSEM 300, Carl Zeiss, Germany).

### 5.15 Molecular docking

The protein data bank (PDB) file of murine GSDMD (6N9N) was downloaded from the RCSB PDB website (https://www.rcsb.org). Water and other ligands in GSDMD were removed by Pymol software. The standard database format files of CA and FA were downloaded from the PubChem website (https://pubchem.ncbi.nlm.nih.gov) and converted into PDB files by Open Babel software. First, the coordinate protein data bank, partial charge, and atom type files of ligands and receptors were created by Autodock tools: adding hydrogen atoms, adding partial charges, assigning atom types, choosing a root atom, defining rotatable bonds, and building the torsion tree. Secondly, the grid maps that make docking calculation fast were calculated by Autogrid: opening macromolecule, setting map types and opening receptor, setting grid boxes, outputting the “.gpf” file, and running. Finally, the ligand‐receptor interaction was evaluated by Autodock: opening macromolecule and ligands, setting search parameters and docking parameters, outputting the “.dpf” file, and running. The docking conformations and binding energies were displayed by Autodock. The photographs shown in this research were optimized by Pymol.

### 5.16 Biotin affinity pull‐down assay

CA and biotin were dissolved in anhydrous *N,N‐*dimethylformamide, and the solution was stirred in dark at room temperature. 20 ml of cold water was added to the reaction mixture, and the extraction of the organic phase was repeated three times. The organic layer was further washed once and then dried with anhydrous Na_2_SO_4_. The mixture was concentrated and subjected to silica gel column chromatography.

J774A.1 cells were lysed with lysis buffer containing a 1% protease inhibitor cocktail as previously described. Pre‐washed streptavidin agarose beads were incubated with free biotin or CA‐biotin at 4°C with rotation and blocked by a biotin‐blocking solution. Subsequently, the cell lysate was added to the system and incubated overnight at 4°C with rotation. The beads were washed three times with elution buffer, then denatured protein was separated by SDS‐PAGE and visualized by western blotting.

### 5.17 Statistical analysis

All data were obtained from at least three independent experiments, analyzed using GraphPad Prism 7, and presented as the mean ± standard error of the mean. Data were performed using one‐way analysis of variance followed by Duncan's test for multiple comparisons and Log‐rank (Mantel‐Cox) test for survival curve analysis. The *p*‐values < 0.05 were considered statistically significant for all studies.

## AUTHOR CONTRIBUTIONS

Mingjiang Liu, Xiaolong Xu, Jingui Li, and Qingquan Liu participated in the design of the study. Mingjiang Liu, Chenglong Yu, and Dandan Liu performed the experiments. Huahao Fan, Chi Zhang, Xin Zhao, Shasha He, Hui Jiang, and Yuhong Guo provided intellectual input. Chenglong Yu, Huiwen Wang, and Xuerui Wang analyzed the data. Mingjiang Liu, Dandan Liu, and Minxia Zhang wrote the manuscript. Xiaolong Xu, Ruonan Bo, and Jingui Li reviewed and prepared the final version of the manuscript. All authors discussed the ideas for this study and approved the final manuscript.

## CONFLICT OF INTEREST STATEMENT

The authors declare no conflict of interest.

## ETHICS STATEMENT

All animal experiments were conducted under the Animal Care and Use Committee of Yangzhou University. All experimental animal ethics are approved by the Experimental Animal Ethics Committee of Yangzhou University (No. 202103‐008).

## Data Availability

The data used to support the findings of this study are available from the corresponding author upon request.
